# Evaluation of naturally occurring IgG anti-Vi antibody titers as predictors and correlates of typhoid fever in Dhaka, Bangladesh

**DOI:** 10.1186/s12879-025-10548-8

**Published:** 2025-01-31

**Authors:** Farhana Khanam, Natasha Y. Rickett, Faisal Ahmmed, Md Taufiqul Islam, Cecilia Kathure Mbae, Justin Im, Asma Binte Aziz, Beatrice Ongadi, Fahima Chowdhury, Ashraful Islam Khan, Afroza Akter, Md Golam Firoj, Sadia Isfat Ara Rahman, Kassa Haile, Se Eun Park, Martin Bundi Mwebia, Moses Mwangi, Benjamin Ngugi, Meseret Gebre Behute, Kelvin Kering, Suneth Agampodi, Suman Kanungo, Xinxue Liu, Andrew J. Pollard, K. Zaman, Deok Ryun Kim, Samuel Kariuki, Firdausi Qadri, John D. Clemens

**Affiliations:** 1 International Centre for Diarrhoeal Disease Research, Dhaka, Bangladesh; 2 International Vaccine Institute, Clinical, Assessment, Regulatory, Evaluation Unit (CARE Unit), Seoul, Republic of Korea; 3 Kenya Medical Research Institute (KEMRI), Nairobi, Kenya; 4 Research Investment for Global Health Technology (RIGHT) Foundation, Seoul, Republic of Korea; 5 Armauer Hansen Research Institute, Addis Ababa, Ethiopia; 6 Department of Global Health and Disease Control, Yonsei University Graduate School of Public Health, Seoul, Republic of Korea; 7 ICMR- National Institute for Research in Bacterial Infections, Kolkata, India; 8 Oxford Vaccine Group, Department of Paediatrics, University of Oxford, Oxford, UK; 9 NIHR Oxford Biomedical Research Centre, Oxford University Hospitals NHS Foundation Trust, Oxford, UK; 10 UCLA Fielding School of Public Health, Los Angeles, CA 90095-1772 USA

**Keywords:** Bangladesh, IgG anti-Vi antibodies, Natural protection, Typhoid fever

## Abstract

**Background:**

When delivered through vaccination Vi-polysaccharide antigen of *Salmonella enterica* serotype Typhi protects against typhoid by inducing IgG anti-Vi antibodies. We aimed to determine whether the presence of antibodies following natural infection is associated with a lower incidence of typhoid fever in endemic regions.

**Methods:**

We analyzed data from a cohort study of typhoid fever conducted in Dhaka, Bangladesh. Plasma IgG anti-Vi antibodies were measured using a standard enzyme-linked immunosorbent assay in random serosurveys of a population that had not previously received typhoid vaccination. Participants were followed for up to 20 months for culture-confirmed typhoid fever. The receiver operating characteristic (ROC) curve and Cox proportional hazard models were used to evaluate the associations between antibody levels and typhoid risk.

**Results:**

The ROC analysis revealed that IgG anti-Vi antibody titers were predictive of typhoid risk among the 8,261 serosurvey participants (area under the curve: 0·63; 95% confidence interval (CI): 0·58─0·67). Detection of any antibodies was associated with a lower risk of typhoid in crude analyses (hazard ratio (HR): 0·13; 95% CI: 0·03─0·52), though this association declined after adjustment (HR: 0·32; 95% CI: 0·07─1·40). A positive correlation was observed between IgG anti-Vi titers and age (correlation coefficient 0·35; *p* < 0·001), with a stepwise increase in detectable antibodies by age, ranging from 12·0% (280/2,333) in children under 5 years to 54·2% (463/854) in individuals 50 years and older (*p* < 0·001).

**Conclusions:**

In typhoid-endemic populations, IgG anti-Vi antibodies may indicate natural immunity to typhoid. The increasing seroprevalence with age suggests that these antibodies could serve as markers of cumulative past typhoid infections. Future research should explore these possibilities.

**Clinical trial number:**

Not applicable.

## Background

The human-restricted pathogen *Salmonella enterica* serovar Typhi (*Salmonella* Typhi), transmitted through oral ingestion of contaminated food or water, causes typhoid fever. It is estimated that in 2019 there were 9 million typhoid fever cases, leading to 110,000 deaths, mostly in low- and middle-income countries in Asia and Africa [1].

The Vi capsular polysaccharide antigen is an essential virulence factor of *Salmonella* Typhi. The Vi antigen is highly immunogenic and has been used to develop effective vaccines consisting of both Vi polysaccharide (Vi-PS) alone and protein-Vi typhoid conjugate vaccines (TCVs) [2–5]. Systemic IgG anti-Vi antibodies are the mediators of protection by these vaccines [6]. However, serum or plasma anti-Vi antibodies following natural typhoid infections in unvaccinated persons appear to be more modest in magnitude, and it is not known whether titers of such antibodies measured in endemic settings correlate with a lower future risk of typhoid fever [7]. This uncertainty is underscored by observations that protection by an episode of typhoid fever against recurrent typhoid is modest in magnitude [8] and that the highest natural titers are seen in patients with chronic typhoid shedding [9]. It is also not known whether the seroprevalence of these antibodies might serve as a marker of the magnitude of past typhoid infections in unvaccinated populations from typhoid-endemic regions. In this study, we evaluated whether naturally acquired detectable levels of IgG anti-Vi antibodies in persons in a typhoid-endemic area who had not received typhoid vaccine were associated with a lower future risk of typhoid fever. We also explored whether the prevalence and magnitude of these antibodies increase with age and correlate with other risk factors for typhoid, as would be expected if they served as markers of the cumulative burden of antecedent typhoid infections in the population.

## Methods

### Study setting and ethical approval

As described in detail elsewhere, the Strategic Typhoid Alliance across Africa and Asia (STRATAA) study was a prospective, longitudinal study with facility-based passive surveillance conducted within a censused total population of 111,695 in Mirpur (wards 3 and 5), Dhaka, Bangladesh, to characterize the burden of enteric (typhoid and paratyphoid) fever [10].

The study was conducted under the provisions of the Helsinki Declaration. The study protocol was approved by the research review committee and ethical review committee of the International Centre for Diarrhoeal Disease Research, Bangladesh. Informed written consent was obtained for all study participants, or from a parent or guardian for minors, and informed written assent was obtained from individuals aged 11–17 years. Anonymized data were obtained from all study participants.

### Census activities

In the STRATAA study, a baseline census was performed between June 1, 2016, and August 31, 2016, to enumerate the entire population in the study area, except non-permanent residents and individuals who planned to move out within a month. During the censuses, individual- and household-level socioeconomic and demographic data and household geopositioning coordinates were ascertained after obtaining informed consent.

### Serological surveys for measuring IgG anti-vi response

Age stratified (0–4 years, 5–9 years, 10–14 years, 15–29 years, 30–49 years, and ≥ 50 years), participants were randomly selected from the list of censused population and enrolled in the serosurvey component of the STRATAA study. The strata were designed to reflect the proportional distribution of different age groups as found in the census conducted from March 2017 to February 2018. Apparently, healthy participants residing in the study catchment area with no history of receipt of any typhoid vaccine and no history of blood transfusion within the last three months were approached for enrollment. After obtaining informed consent, the recruitment of selected participants of different age groups was carried out throughout the year in four quarters, comprising three months in each quarter (quarter 1: March 2017–May 2017, *n* = 1,865; quarter 2: June 2017–August 2017, *n* = 2,132; quarter 3: September 2017–November 2017, *n* = 2,143; and quarter 4: December 2017–February 2018, *n* = 2,121). Approximately 3 milliliters (mL) of blood specimens were collected in ethylenediaminetetraacetic acid (EDTA) tubes for carrying out enzyme-linked immunosorbent assay (ELISA). Serosurvey participants who subsequently presented with fever at health facilities were enrolled in the passive surveillance component to determine blood culture-confirmed typhoid fever patients.

### Surveillance for typhoid fever

Surveillance for typhoid was carried out from August 2016 to January 2019 at eight health facilities. Upon presenting for care, the identities of patients were confirmed against computerized census data available in the health facilities. Patients presenting with a history of fever for ≥ 48 h or a measured axillary temperature of ≥ 38·0 °C were eligible for enrollment. Blood specimens (~ 3 mL from patients aged 1–15 years and ~ 5 mL from patients aged ≥ 16 years) were collected from all enrolled patients for microbiological culture to isolate *Salmonella* Typhi. Clinical data on physical examinations were systematically recorded by study physicians. Fever visits in which the onset of fever was < 14 days after discharge from the previous fever visit were concatenated into a single “fever episode”. Fever episodes in which one or more blood cultures were positive for *Salmonella* Typhi were defined as typhoid fever episodes.

### Laboratory methods

Blood culture specimens underwent microbiological evaluation using automated systems such as the BD BACTEC Blood Culture System (Becton-Dickinson, Franklin Lakes, NJ, USA) or BacT/ALERT (BioMerieux, Marcy-l’Étoile, France). When cultures tested positive for growth, *Salmonella* Typhi was identified and confirmed through colony morphology, Gram staining, standard biochemical tests, and specific antisera [10].

For the serosurvey component, plasma was separated from EDTA blood by centrifugation, following which plasma IgG anti-Vi antibody titers were measured using a commercial ELISA kit (VaccZyme; The Binding Site Group Ltd, Birmingham, UK) as per the manufacturer’s instructions [10–12]. The assay’s detection range was 7·4–600 unit/milliliters (U/mL).

### Analysis

All serosurvey participants were followed for typhoid fever from their enrollment, with follow-up censored at the time of death, migration out of the study area, the end of passive surveillance or if typhoid was detected within the follow-up period.

To assess the relationship between age and antibody titres, a weighted regression line of age on anti-Vi IgG titers was estimated, where the loess (locally estimated scatterplot smoothing) curve was used to smooth the regression line. The optimal smoothing parameter was selected from 0.10 to 0.90 by minimizing the bias-corrected Akaike information criterion that strikes a balance between the residual sum of squares and the complexity of the fit [13]. To assess the age-specific pattern of plasma IgG anti-Vi antibody titers, we compared the proportions of persons with detectable levels (≥ 7.4 U/mL) of these antibodies by age, as well as the magnitude of log titers in relation to age.

To assess whether plasma IgG anti-Vi antibodies measured in the serosurveys predicted subsequent typhoid fever, we explored the data using a reverse cumulative distribution (RCD) curve for participants who developed typhoid and those who did not. Samples with values below 7·4 U/mL (the lower limit of detection by the assay) were assigned a value of 3·7 U/mL, while those exceeding 600 U/mL (the upper limit of detection by the assay) were reported as 600 U/mL.

We next constructed a receiver operating characteristic (ROC) curve presenting the sensitivity and specificity for alternative thresholds of plasma IgG anti-Vi antibodies in predicting typhoid fever. The area under the ROC curve (AUC) was calculated to evaluate the overall performance of plasma IgG anti-Vi antibodies in predicting typhoid fever. A perfect predictive relationship would have an AUC of 1, while a random relationship would have an AUC of 0·50. The 95% CI for the AUC and the p value corresponding to the null hypothesis that the AUC is equal to 0.5 were calculated as described elsewhere [14]. A cut-off for the optimal prediction of the titer expressed dichotomously was identified using the Youden index, maximizing the (sensitivity + specificity–1) of the threshold titer under analysis.

Bivariate comparisons of categorical variables were statistically assessed using the chi-square test. For simple comparisons of continuous variables, we used the Student t-test, or the Wilcoxon rank sum test when assumptions of the t-test were not met. Plasma IgG anti-Vi antibody titers were converted into logarithms for these comparisons to render a normal distribution. Spearman correlation coefficients were calculated to evaluate the correlation between continuous variables.

Incidence rates of typhoid fever among serosurvey participants were calculated using the number of typhoid episodes as the numerator and the corresponding person-years of follow-up as the denominator. The 95% confidence intervals (CIs) for incidence rates were estimated using exact Poisson confidence limits, a Chi-square-based formula that provides precise intervals in case of small event counts, by leveraging the relationship between the Poisson and Chi-square distributions [15]. We further analyzed the protection against typhoid associated with IgG anti-Vi antibody titers above versus below this cut-off using Cox proportional-hazards regression models. The time of outcome event in the Cox model referred to the time from study enrollment to the date of the censoring event. In these models, we assessed IgG anti-Vi antibody titers expressed dichotomously, together with age as an independent variable, after confirming first that these independent variables fulfilled the proportionality assumption of the model [16]. To account for household-level clustering of participants, we used a robust sandwich covariance matrix to calculate the confidence interval and p value. Hazard ratios (HRs) and their 95% CIs were calculated by exponentiating the coefficient of the dichotomous IgG anti-Vi antibody variable.

The p values were interpreted in a two-tailed fashion, and those less than 0·05 were considered statistically significant. Statistical analyses were performed using SAS software, Version 9.4 of the SAS System for Windows (SAS Institute Inc., Cary, NC, USA).

## Results

### Assembly of participants

**Fig. 1:**
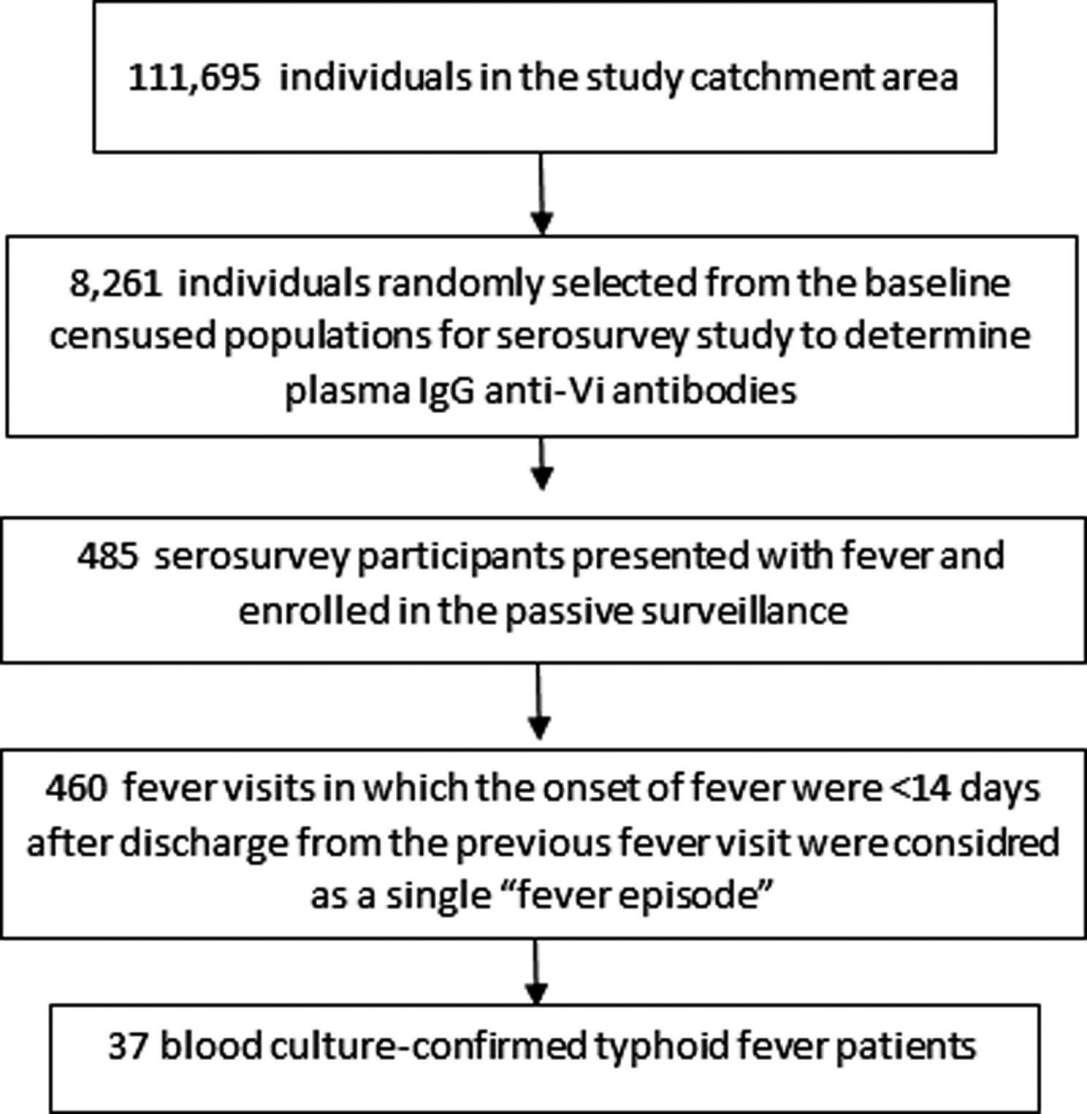
Consort diagram presenting assembly of the fever episodes and typhoid fever cases among serosurvey participants

A total of 111,695 participants were enrolled in the STRATAA study during the census. Among them, 8,261 age-stratified participants were randomly selected for the serosurvey component (2,333 aged 0–4 years, 1,316 aged 5–9 years, 802 aged 10–14 years, 1,168 aged 15–29 years, 1,788 aged 30–49 years and 854 aged ≥ 50 years). After enrollment in the serosurveys, a total of 485 serosurvey participants presented for care of febrile illness at the passive surveillance sites, among whom 37 were blood culture-confirmed typhoid cases (Fig. 1).

### Pattern of antibodies in the serosurveys

**Fig. 2:**
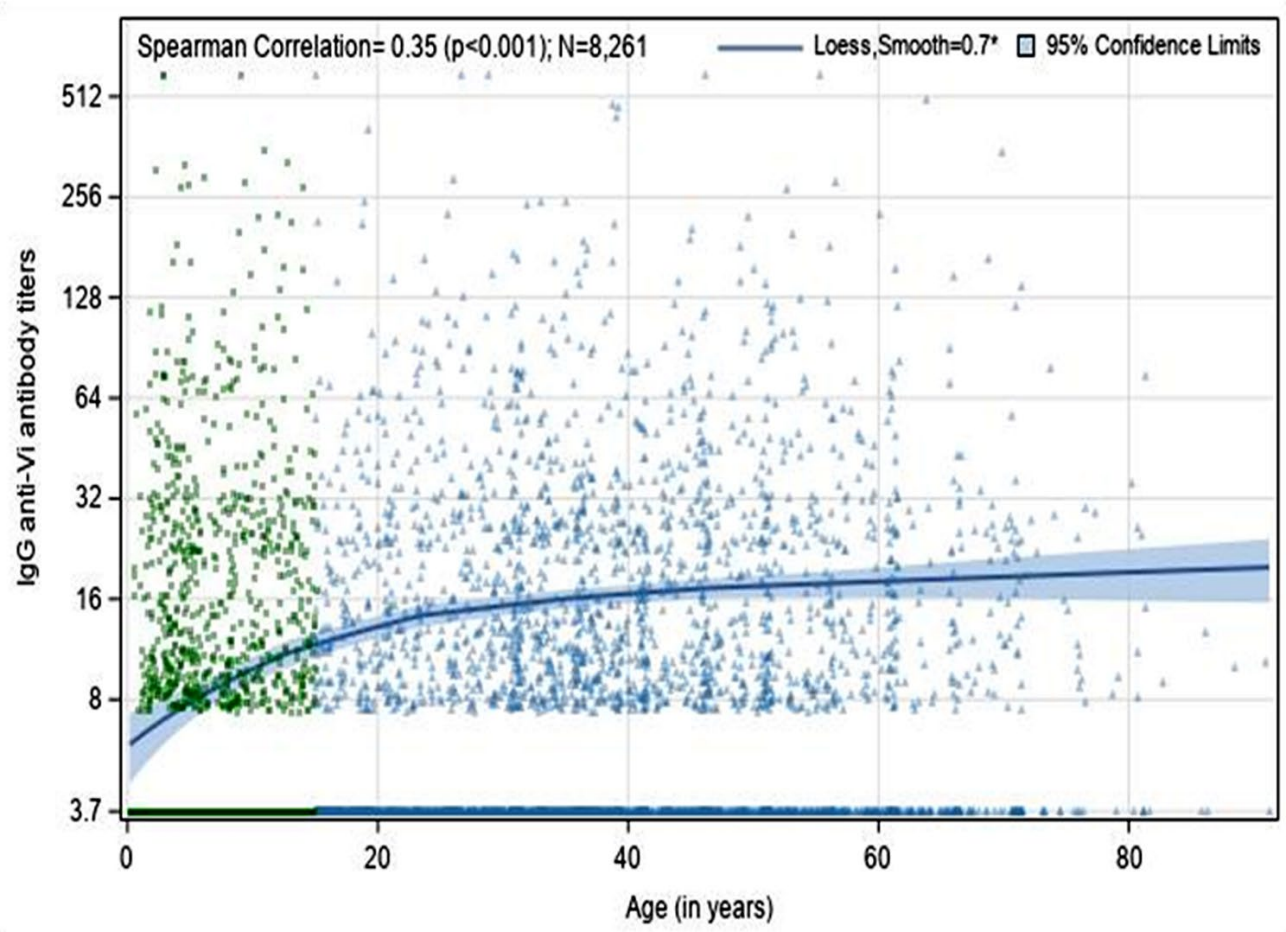
Relationship between ages of serosurvey participants and IgG anti-Vi antibody titers. *Loess (locally estimated scatterplot smoothing), is a nonparametric technique that uses local weighted regression to fit a smooth curve through the points in a scatter plot, as described in the method section. A smoothing parameter value of 0·70, optimally chosen using the bias-corrected Akaike information criterion, ranges from 0·10 to 0·90. The figure presents the fitted localized regression curve using 70% of the neighboring data points

**Fig. 3:**
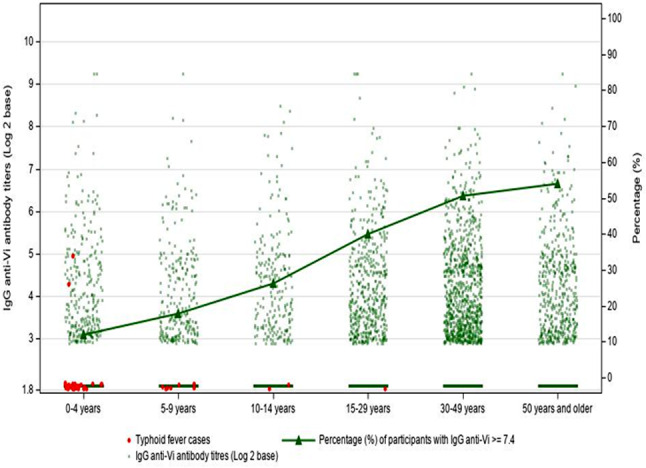
Proportion of participants with delectable levels of IgG anti-Vi antibody titers among different age groups

Among 8,261 individuals, 2,566 (31·1%) had IgG anti-Vi antibody titers at or above the lower limit of detection (7·4 U/mL) of the assay, including 1,837 (48·2%) individuals aged 15 years and older who had the antibody titers at or above the same lower detectable limit (Table 1). We observed a positive correlation between IgG anti-Vi titers and age in years (correlation coefficient was 0·35; *p* < 0·001) (Fig. 2). When analyzed by age group, we found that seropositivity rates were 12·0% for ages 0–4 years, 18·0% for ages 5–9 years, 26·4% for ages 10–14 years, 40·1% for ages 15–29 years, 50.7% for ages 30–49 years, and 54·2% for ages 50 years and older (Fig. 3).

**Table 1:** Baseline characteristics of serosurvey participants stratified by IgG anti-vi titers

Variables	IgG anti-Vi < 7·4 U/mL (N^*^=5,695)	IgG anti-Vi ≥ 7·4 U/mL (N^*^=2,566)	Total (N^*^=8,261)	*p* value^†^
**IgG anti-Vi titers (U/mL)**				
Geometric Mean (Geometric SD^‡^)	3·7 (1·0)	18·8 (2·3)	6·1 (2·4)	< 0·001
Median (Q1^**§**^– Q3^**¶**^)	3·7 (3·7–3·7)	15·1 (9·6–29·0)	3·7 (3·7–8·9)	< 0·001
**Age (year) at baseline serosurvey**				
Mean (SD^‡^)	16·4 (17·4)	30·6 (19·2)	20·8 (19·1)	< 0·001
Median (Q1^**§**^– Q3^**¶**^)	8·4 (4·0–26·1)	31·2 (13·0–45·2)	12·9 (5·0–35·7)	< 0·001
**Age groups– n (%)** ^**^				
15 years and older	1,973 (34·6%)	1,837 (71·6%)	3,810 (46·1%)	< 0·001
**Sex– n (%)** ^**^				
Male	2,797 (49·1%)	1,230 (47·9%)	4,027 (48·7%)	0·32
**Household head education– n (%)** ^**^				
Some education	3,716 (65·3%)	1,576 (61·4%)	5,292 (64·1%)	< 0·001
**Household head occupation– n (%)** ^**^				
Paid employees (paid domestic workers or self-employed)	4,855 (85·3%)	2,114 (82·4%)	6,969 (84·4%)	< 0·001
**Serosurvey enrollment quarters– n (%)** ^**^				
Quarter 1^**††**^	1285 (22·6%)	580 (22·6%)	1865 (22·6%)	0·52
Quarter 2^‡‡^	1472 (25·8%)	660 (25·7%)	2132 (25·8%)	
Quarter 3^**§§**^	1454 (25·5%)	689 (26·9%)	2143 (25·9%)	
Quarter 4^**¶¶**^	1484 (26·1%)	637 (24·8%)	2121 (25·7%)	

^*^N: total number of participants;

^**†**^p value has been derived using a two-sample t-test and Wilcoxon rank-sum test for mean and median comparison– respectively for continuous variables– and chi-square test for binary variables;

^‡^SD: Standard deviation;

^**§**^Q1: first quartile;

^**¶**^Q3: third quartile;

^**^n(%): number (percent) of participants;

^**††**^Quarter 1 (March 2017–May 2017);

^‡‡^Quarter 2 (June 2017–August 2017);

^**§§**^Quarter 3 (September 2017–November 2017)

^**¶¶**^Quarter 4 (December 2017–February 2018)

### Evaluation of the performance of plasma IgG anti-vi titers in predicting typhoid

**Fig. 4:**
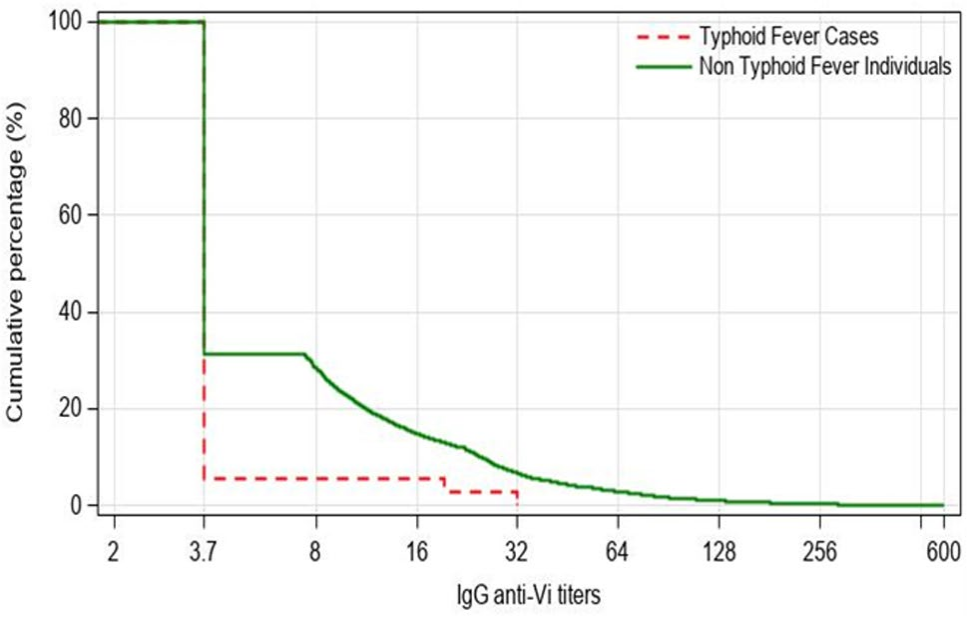
Reverse cumulative distribution curve of IgG anti-Vi antibody titers by culture-confirmed typhoid fever status. The graph’s horizontal axis represents the IgG anti-Vi antibody titers on a logarithmic scale, and the vertical axis represents the percentage of participants with at least that level of antibody titers, ranging from 0% at the highest level to 100% at the lowest level. Samples with values below 7·4 U/mL (the lower limit of detection by the assay) were assigned a value of 3·7 U/mL and those exceeding 600 U/mL (the upper limit of detection by the assay) were reported as 600 U/mL

Figure 4 illustrates RCD curve for titers of IgG anti-Vi antibodies at the time of the serosurveys. The curve provided a visual depiction of whether a protective relationship between antibodies and the risk of typhoid fever existed. The RCD curve for the 37 participants who developed typhoid was lower than that for the rest of 8,224 participants who did not throughout the range of detectable antibody titers, indicative of a protective relationship between IgG anti-Vi antibody titers and typhoid risk. No serosurvey participants who developed typhoid had a titer above 32 U/mL.

**Fig. 5:**
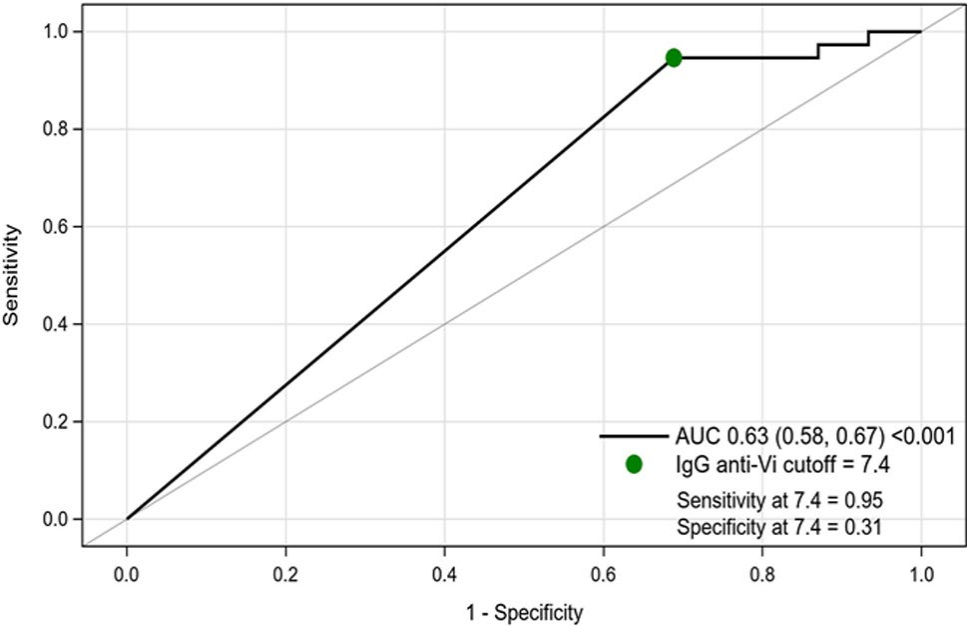
Receiver operating characteristic curve for plasma IgG anti-Vi antibody titers in predicting subsequent typhoid fever with the area under the curve (AUC). The green dot denotes the sensitivity and specificity at the cut-off of 7.4 for IgG anti-Vi antibody titers using the Youden index to detect typhoid fever. The Youden index is calculated as the maximum of (sensitivity + specificity– 1)

Figure 5 demonstrates the ROC curve describing the sensitivity and (1- specificity) of using progressively higher thresholds of baseline titers in predicting typhoid. The ROC curve was significantly above the line reflecting no predictive relationship (AUC: 0·63; *p* < 0·001; 95% CI: 0·58─0·67). The cut-off for creating a dichotomous predictive titer, maximizing the Youden index, was 7·4 U/mL, the lower limit of detection of antibodies by the assay, with a corresponding sensitivity and specificity of 0·95 and 0·31.

In participants with IgG anti-Vi titers of ≥ 7·4 U/mL, the incidence of typhoid fever was 0·61 per 1,000 person-years (95% CI: 0·15─2·42). In participants without detectable antibody titers (< 7·4 U/mL), the incidence rate was 4·79 per 1,000 person-years (95% CI: 3·44─6·66). The crude hazard ratio (HR) comparing the hazards between individuals with IgG anti-Vi titers of ≥ 7·4 U/mL versus < 7·4 U/mL was 0·13 (95% CI: 0·03─0·52; *p* = 0·004). Adjusting for age, the household level correlation of disease incidence, and the serosurvey quarters of enrollment, the adjusted HR was 0·32 (95% CI: 0·07─1·40; *p* = 0·130) (Table 2). The adjusted HR was nearly identical when the analysis was restricted to subjects aged < 15 years at the time of bleeding (HR = 0·36; 95% CI: 0·08─1·53; *p* = 0·170).

**Table 2:** Crude and adjusted hazard ratios for the associations between plasma IgG anti-vi antibody titers and the rate of typhoid

IgG anti-Vi antibody titers	Typhoid cases; total number of participants	Person-years follow-up	Incidence per 1,000 person-years (95% CI)	Crude			Adjusted^†^		
					Hazard ratio (95% CI*)	*p* value	Hazard ratio (95% CI*)		*p* value
***All Participants***									
< 7·4 U/mL	35; 5,695	7,314	4·79 (3·33 − 6·65)		Ref.		Ref.		
≥ 7·4 U/mL)	2; 2,566	3,304	0·61 (0·07 − 2·19)		0·13 (0·03─0·52)	0·004	0·32 (0·07─1·40)	0·13	
***< 15 years old***									
< 7·4 U/mL	34; 3,722	4,799	7·08 (4·91 − 9·90)		Ref.		Ref.		
≥ 7·4 U/mL)	2; 729	951	2·10 (0·26 − 7·60)		0·30 (0·07─1·24)	0·096	0·36 (0·08─1·53)		0·17

*CI: Confidence Interval. The CIs for incidence rates were calculated as the estimated exact Poisson confidence limits. CIs of hazard ratios were derived from the Cox proportional hazard model with a robust sandwich covariance matrix to account for household-level clustering of participants;

^**†**^Estimates were derived from Cox proportional hazards models, adjusted for the survey enrollment design variable, within-household correlation, and age at baseline serosurvey

### Correlates of plasma IgG anti-vi antibodies

The mean age was 30·6 years for participants who had plasma IgG anti-Vi titers ≥ 7·4 U/mL, and 16·4 years for participants who did not have detectable antibodies (*p* < 0·001) (Table 1). We did not observe any significant relationship between sex and IgG anti-Vi titers. However, lower socioeconomic status, as reflected by the education level and occupation of household heads, appeared to be associated with higher IgG anti-Vi titers. There were no significant differences between IgG anti-Vi titers and enrollment in different serosurveys, indicating that the observed differences in IgG anti-Vi titers were not influenced by the timing of enrollment of serosurvey participants in the study.

## Discussion

We evaluated the presence and titers of plasma IgG anti-Vi antibodies in randomly selected individuals from a population with endemic typhoid and followed them prospectively to detect subsequent cases of typhoid fever. Our results found a strong inverse association between the level of such antibodies, using a standard assay, and the risk of typhoid, as indicated by ROC analysis (AUC = 0·63; 95% CI: 0·58─0·67; *p* < 0·001). After the dichotomization of titers into detectable versus undetectable titers, the presence of these antibodies was strongly and inversely related to the hazard of typhoid in crude analyses ((HR = 0·13 (95% CI: 0·03─0·52); *p* = 0·004). However, this inverse association lost statistical significance in analyses controlling for potential confounding variables ((HR = 0·32 (95% CI: 0·07─1·40); *p* = 0·130). The point estimate of the hazard ratio remained strong in this multivariable analysis raises the possibility that the study lacked adequate power to evaluate this inverse relationship possibly due to an insufficient number of outcome events. Additionally, no person who developed typhoid had a titer greater than 32 U/mL, raising the possibility of an absolute protective titer. The presence and magnitude of these antibodies increased with age, indicating that they could reflect the cumulative level of past typhoid infection. Concordant with this interpretation, the titers of these antibodies were higher among individuals residing in households of lower socioeconomic status, which are known to have a higher risk of typhoid in this endemic setting.

Our study was strengthened by the conduct of random, population-based serosurveys; the use of a well-standardized and WHO-recommended immunoassay for evaluation of plasma IgG anti-Vi antibodies; and prospective and systematic surveillance for blood culture-confirmed typhoid fever. However, as already noted, our analysis was based on detecting only 37 typhoid cases among participants in the serosurveys and thus had limited power to evaluate protective relationships. Moreover, follow-up of individuals for the development of typhoid was only up to 20 months, precluding assessment of longer-term protective associations. Additionally, the study was carried out in a population with a high prevalence of typhoid, which may restrict the generalizability of these findings to populations with a lower incidence of typhoid infection.

The observed inverse relationship is interesting since plasma IgG anti-Vi titers have historically been measured to detect very high levels, known markers of chronic typhoid carriage. Historically, this relationship created great skepticism about whether the Vi-polysaccharide (Vi-PS) antigen was a protective antigen, a misconception, that was refuted by the initial demonstration of the efficacy trial of the Vi-PS vaccine in Nepal [17]. The fact that Vi-PS is a demonstrably protective antigen adds plausibility to our findings that seroprevalent IgG anti-Vi antibodies are associated with protection against future typhoid.

That said, it was surprising that the mere presence of these antibodies, as measured by this assay, appeared to be highly protective, although the relationship was not statistically significant in multivariable analyses. Substantial increases in antibody titers are considered necessary for high-level protection after receipt of either Vi-PS or Vi-protein conjugate vaccines, though protective titers have not been evaluated using this assay in these studies [11, 12]. This discordancy may point to the fact that antibodies measured in our study originated from previous typhoid infection, rather than receipt of Vi-PS per se. It also raises the possibility that the antibodies detected in our study may reflect multiple immune responses to typhoid infection, including both humoral and cell-mediated responses.

Though both *Citrobacter freundii* and *Salmonella* Paratyphi C express the Vi-PS antigen [18], *Citrobacter freundii* is an opportunistic pathogen [19] and *Salmonella* Paratyphi C is not commonly isolated in this community [10], making it most likely that the IgG anti-Vi antibodies demonstrated in this endemic population resulted from earlier exposure to *Salmonella* Typhi. The possible protective relationship observed in our study is also interesting because the protection conferred by natural typhoid against recurrent typhoid has been shown in limited studies to be modest in magnitude [8]. In addition, a study using the controlled human infection model of *Salmonella* Typhi with the Vi-PS and Vi-tetanus toxoid conjugate vaccines found that both the total quantity and functionality of IgG anti-Vi antibodies are crucial for providing protection against typhoid fever [11]. A challenge model setting is unable to replicate many characteristics of a natural *Salmonella* Typhi infection due to variations in factors such as the *Salmonella* Typhi strain, the challenge inoculum, the time gap between vaccination and challenge, and differences in research population (e.g., children vs. adults), therefore, it is necessary to carry out studies in the context of natural *Salmonella* Typhi infection [11]. If our findings are confirmed by future studies, they may suggest that the detection of plasma IgG anti-Vi antibodies may serve as a useful marker of past typhoid infection and may be valuable for assessing the endemicity of typhoid through simple seroprevalence surveys. This is particularly important because assessing the burden of typhoid through longitudinal surveillance of large populations, a rare disease, is expensive, logistically complex, and difficult to organize.

## Conclusions

In conclusion, our findings raise the possibility that serosurveys of plasma IgG anti-Vi antibodies in populations with endemic typhoid may prove to be a useful tool for characterizing the natural protective immune status of populations as well as for providing evidence on the cumulative burden of typhoid experienced by these populations. As useful immunological measurements for serosurveys are currently lacking for either purpose, future research is needed to further evaluate the preliminary findings provided by this study.

## Data Availability

A de-identified analytical data set will be made available upon requests directed to the Institutional Review Board (IRB) coordinator, of the icddr, b, M.A Salam Khan, at salamk@icddrb.org, or at info@icddrb.org. Only after approval of a proposal data can be shared through a secure online platform. Approval of the proposal will be subject to scientific review by the institutional review board at icddr, b. Sharing of data will also be subject to the published data access rules of the icddr, b. The requestor will need to sign a standard data access agreement required by the icddr, b.

## Abbreviations

AUC: Area Under the Curve
CI: Confidence Interval
EDTA: Ethylenediaminetetraacetic Acid
ELISA: Enzyme-linked Immunosorbent Assay
HR: Hazard Ratio
mL: Milliliters
PS: Polysaccharide
RCD: Reverse Cumulative Distribution
ROC: Receiver Operating Characteristic
STRATAA: Strategic Typhoid Alliance Across Africa and Asia
TCV: Typhoid Conjugate Vaccines

## References

[1] (IHME) IfHMaE. Global Burden of Disease Collaborative Network. GBD 2019 Cause and Risk Summaries: Typhoid fever 2019 [Available from: http://www.healthdata.org/gbd/2019

[2] Sur D, Ochiai RL, Bhattacharya SK, Ganguly NK, Ali M, Manna B, et al. A cluster-randomized effectiveness trial of vi typhoid vaccine in India. N Engl J Med. 2009;361(4):335–44. 19625715 10.1056/NEJMoa0807521

[3] Mohan VK, Varanasi V, Singh A, Pasetti MF, Levine MM, Venkatesan R, et al. Safety and immunogenicity of a vi polysaccharide-tetanus toxoid conjugate vaccine (Typbar-TCV) in healthy infants, children, and adults in typhoid endemic areas: a multicenter, 2-cohort, open-label, double-blind, randomized controlled phase 3 study. Clin Infect Dis. 2015;61(3):393–402. 25870324 10.1093/cid/civ295

[4] WHO. Prequalification of Medical Product 2017 [cited 2024 18 July]. Available from: https://extranet.who.int/prequal/vaccines/p/typbar-tcv-0

[5] K. N. More typhoid conjugate vaccines, more impact 2020 [Available from: https://www.coalitionagainsttyphoid.org/moretyphoidconjugatevaccines/

[6] Qadri F, Khanam F, Liu X, Theiss-Nyland K, Biswas PK, Bhuiyan AI, et al. Protection by vaccination of children against typhoid fever with a Vi-tetanus toxoid conjugate vaccine in urban Bangladesh: a cluster-randomised trial. Lancet. 2021;398(10301):675–84. 34384540 10.1016/S0140-6736(21)01124-7 PMC8387974

[7] House D, Ho VA, Diep TS, Chinh NT, Bay PV, Vinh H, et al. Antibodies to the vi capsule of Salmonella Typhi in the serum of typhoid patients and healthy control subjects from a typhoid endemic region. J Infect Dev Ctries. 2008;2(4):308–12. 19741294 10.3855/jidc.227

[8] Im J, Islam MT, Kim DR, Ahmmed F, Chon Y, Zaman K, et al. Protection conferred by typhoid fever against recurrent typhoid fever in urban Kolkata. PLoS Negl Trop Dis. 2020;14(8):e0008530. 32804950 10.1371/journal.pntd.0008530 PMC7430703

[9] Lagos RM, Sikorski MJ, Hormazábal JC, Fernandez A, Duarte S, Pasetti MF et al. Detecting residual chronic Salmonella Typhi carriers on the road to typhoid elimination in Santiago, Chile, 2017–2019. J Infect Dis. 2023. 10.1093/infdis/jiad585 PMC11326835 38123455

[10] Meiring JE, Shakya M, Khanam F, Voysey M, Phillips MT, Tonks S, et al. Burden of enteric fever at three urban sites in Africa and Asia: a multicentre population-based study. Lancet Glob Health. 2021;9(12):e1688–96. 34798028 10.1016/S2214-109X(21)00370-3 PMC8609278

[11] Jin C, Gibani MM, Moore M, Juel HB, Jones E, Meiring J, et al. Efficacy and immunogenicity of a Vi-tetanus toxoid conjugate vaccine in the prevention of typhoid fever using a controlled human infection model of Salmonella Typhi: a randomised controlled, phase 2b trial. Lancet. 2017;390(10111):2472–80. 28965718 10.1016/S0140-6736(17)32149-9 PMC5720597

[12] Jin C, Hill J, Gunn BM, Yu W-H, Dahora LC, Jones E, et al. Vi-specific serological correlates of protection for typhoid fever. J Exp Med. 2020;218(2):e20201116. 10.1084/jem.20201116 PMC7668386 33180929

[13] Hurvich CM, Simonoff JS, Tsai C-L. Smoothing parameter selection in nonparametric regression using an improved Akaike information criterion. J Royal Stat Soc Ser B: Stat Methodol. 1998;60(2):271–93.

[14] DeLong ER, DeLong DM, Clarke-Pearson DL. Comparing the areas under two or more correlated receiver operating characteristic curves: a nonparametric approach. Biometrics. 1988:837–45. 3203132

[15] Ulm K. Simple method to calculate the confidence interval of a standardized mortality ratio (SMR). Am J Epidemiol. 1990;131(2):373–5. 2296988 10.1093/oxfordjournals.aje.a115507

[16] Lin DY, Wei L-J, Ying Z. Checking the Cox model with cumulative sums of martingale-based residuals. Biometrika. 1993;80(3):557–72.

[17] Acharya IL, Lowe CU, Thapa R, Gurubacharya VL, Shrestha M, Cadoz M, et al. Prevention of typhoid fever in Nepal with the vi capsular polysaccharide of Salmonella typhi. N Engl J Med. 1987;317(18):1101–4. 3657877 10.1056/NEJM198710293171801

[18] Daniels EM, Schneerson R, Egan WM, Szu SC, Robbins JB. Characterization of the Salmonella paratyphi C vi polysaccharide. Infect Immun. 1989;57(10):3159–64. 2506132 10.1128/iai.57.10.3159-3164.1989 PMC260784

[19] Liu H, Zhao Z, Xue Y, Ding K, Xue Q. Fatal cases of Citrobacter freundii septicemia and encephalitis in sheep. J Vet Diagn Invest. 2018;30(2):245–8. 29105585 10.1177/1040638717731090 PMC6505869

